# Self-directed learning and the student learning experience in undergraduate clinical science programs: a scoping review

**DOI:** 10.1007/s10459-024-10383-7

**Published:** 2024-10-23

**Authors:** Ashleigh Finn, Caitlin Fitzgibbon, Natalie Fonda, Cameron M Gosling

**Affiliations:** 1 Department of Paramedicine, Monash University, Level 2, Building H, Peninsula Campus, McMahons Road, Frankston, VIC 3199 Australia; 2 School of Nursing, Midwifery and Paramedicine, Australian Catholic University, 115 Victoria Parade, Fitzroy, VIC 3065 Australia

**Keywords:** Self-directed learning, Clinical science programs, Undergraduate education, Health education, Curriculum, Learning experience

## Abstract

Health professional organisations are increasingly promoting the use of self-directed learning. Furthermore, the rapidly evolving field of healthcare has meant that there is greater emphasis within tertiary education for students to become self-directed learners and possess the skills to engage in life-long learning. The aim of this scoping review was to explore the drivers that improve the student learning experience, in undergraduate clinical science programs that utilise self-directed learning. The Joanna Briggs Institute Scoping Review Methodology guided this study. The electronic databases MEDLINE, Embase, Emcare, Scopus and ERIC were comprehensively searched in April 2022 and re-run August 2023, for peer-reviewed research articles published in English. The original search was developed in MEDLINE and then adapted to each database. Following the Joanna Briggs Scoping Review methodology, articles were screened first by title and abstract and then by full text. Included articles were assessment for methodological quality. The search strategy yielded 2209 articles for screening. 19 met the inclusion criteria. Five key factors were identified which improve the student learning experience in self-directed learning: (i) curricular elements; (ii) educator influence; (iii) impact of peers, (iv) environment; and (v) clinical placement experiences. There are many curricular, environmental, and external factors which can improve the student learning experience in programs that utilise self-directed learning. Greater understanding of these factors will allow educators within clinical science programs to implement self-directed learning strategies more effectively within curriculum.

## Introduction

Self-directed learning (SDL) has become a widely adopted andragogical approach within tertiary curricula. This is especially pertinent in clinical science programs where SDL is promoted within health professional organisations (Wong et al., 2021). Currently, there is greater expectation that students are more independent and accountable for their learning (Shirazi et al., 2017; Wong et al., 2021). However, the assumption that students are already effective self-directed learners, may be misguided. Students are not intrinsically self-directed, (du Toit-Brits, 2019) and for some students, the requirement to be self-directed in their education may be a significant contrast from their prior educational experiences (Choi et al., 2014; Sharples & Moseley, 2010). It has also been suggested that educators may find the implementation of SDL within curriculum difficult, particularly regarding the level of input and specific guidance related to feedback (Alotaibi, 2016; Hewitt-Taylor, 2001; Lunyk-Child et al., 2001).

SDL was first defined by early educational theorist Malcom Knowles (Knowles, 1975) as “a process in which individuals take the initiative, with, or without the help of others, to diagnose their learning needs, formulate learning goals, identify resources for learning, select and implement learning strategies, and evaluate learning outcomes”. SDL is a vital component in Knowles’ theory of ‘andragogy’, an adult learning theory which focuses on a student-centred approach in education (Knowles, 1975).

Acquiring content knowledge alone is inadequate to develop crucial skills of critical thinking, problem-solving and lifelong learning (Kan’an & Osman, 2015). Furthermore, SDL is primarily a learning technique that promotes higher order cognitive skills and increases self-efficacy of the students (Khiat, 2015). The relationship between characteristics such as cognition, motivation, and behaviour, and essential nursing characteristics has been highlighted in the literature (Kuiper et al., 2010). Active involvement in SDL also makes students more flexible, proficient, resourceful and qualified in their education needs, (Shirazi et al., 2017) capable of becoming lifelong learners (du Toit-Brits & Zyl, 2017). This prepares students to be engaged in continued education and meet industry requirements.

Whilst the ability to engage in lifelong learning is an important skill sought by industry bodies, a key competency in the professional practice across many disciplines is the ability to predict an outcome and respond effectively, using reflective and problem-solving skills (Paramedicine Board of Australia, 2021; Nursing & Midwifery Board of Australia, 2016). Therefore, SDL is synonymous with professional practice learning. Professional organisations are increasingly promoting the use of SDL (Wong et al., 2021). The Nursing and Midwifery Council in the United Kingdom, perceive that SDL is a critical component of undergraduate nursing education (Pryce-Miller & Serrant, 2019). Similar sentiments have also been echoed in various other accrediting bodies and professional organisations across the United States of America, including the Liaison Committee on Medial Education (LCME), Accreditation Council for Graduate Medical Education (ACGME) and The American Board of Internal Medicine (ABIM) (Murad & Varkey, 2008). The Medical Council of India recently introduced SDL as a crucial competency element of the undergraduate medical curriculum, outlining specific hours of SDL for all medical students (Agrawal & Verma, 2020).

Research by Smedley (2007) found that SDL was an integral factor driving individual learning to pursue more effective patient care, in undergraduate nursing students in Australia. By exploring more self-directed ways of learning, such as through independent practice, nursing students have also been found to develop necessary qualities, such as responsibility and assertiveness (Bastable, 2008; Mulube & Jooste, 2014). Due to the continued expansion and dynamic nature of knowledge and practice within health care, there is a growing emphasis for students to become lifelong learners (O’Shea, 2003; Wong et al., 2021). Students therefore need to be provided with the tools to be self-directed in their continual education and development (Dunham, 2018; O’Shea, 2003).

Previous research has investigated the influence of personality traits, gender, culture, and sociodemographics on students’ SDL ability and readiness (El-Gilany & Abusaad, 2013; Örs, 2018; Yang et al., 2021; Yuan et al., 2012). Females have a higher degree of readiness to engage in SDL than males (Slater et al., 2017). SDL readiness has also been found to increase with age and higher levels of previous education (Slater et al., 2017; Yuan et al., 2012).

However, there is limited research on the external factors that can impact on the student learning experience. It is therefore imperative to cultivate awareness of appropriate curricula approaches for SDL within tertiary education which ultimately promotes lifelong learning. The aim of this scoping review was to explore the drivers that improve the student learning experience in undergraduate clinical science programs that utilise SDL. The results can aid academics in future curriculum development and ensure that SDL is implemented effectively and appropriately.

## Methods

### Study design

To meet the aim of this scoping review, research studies that met the following participants, concept and context (PCC) search strategy were considered for inclusion:

*Participants:* Students studying in an undergraduate clinical science program.

*Concept:* Self-directed learning.

*Context:* Any curricular, environmental, or external factor which improves the learning experience for the participants.

A scoping review was identified as the most appropriate method for our aim, as it explored key factors related to the research area and identified knowledge gaps (Peters et al., 2020). The Joanna Briggs Institute (JBI) Scoping Review Methodology (Peters et al., 2020) guided this study and findings were reported according to the Preferred Reporting Items for Systematic Reviews and Meta-Analysis Extension for Scoping Reviews (PRISMA-ScR) guidelines (Tricco et al., 2018). A review protocol was developed and was published on Open Science Framework (registration number: 10.17605/OSF.IO/Y3TE8).

### Search strategy

Academic literature was comprehensively searched between 22 and 28 April 2022, using five electronic peer-reviewed databases: MEDLINE, Embase, Emcare, Scopus and ERIC. This search was re-run on 15 August 2023, to ensure currency of the included articles. No date range filters were set. The search strategy utilised a combination of terms based on alternative terms, synonyms for the key words and key concepts (Tables 3 and 4). One author (AF) conducted the literature search and imported the results into Covidence (Covidence, Melbourne Australia).

### Inclusion/exclusion criteria

Literature was eligible for inclusion if it met the following criteria: involved undergraduate students within clinical science programs, the central intervention or outcome was SDL within such programs, the study outcomes included factors which influenced the student learning experience, and study design was quantitative, qualitative, or mixed method. Grey literature was eligible for inclusion. A study was excluded if the article had an unsuitable study design (such as systematic reviews or editorials), was not written in English language or full text access was unavailable.

### Source selection

The citations were exported to EndNote (version 9, Clarivate Analytics, US) and then into Covidence for review. Duplicates were removed and articles underwent independent title and abstract screening, followed by full text screening for eligibility by two authors (AF, CF). Conflicts were resolved by a third author (CG). Reviewers were not blinded to any components of the eligible studies. Once the accepted studies were confirmed, forward and backward citation searching was used to search for additional relevant articles. Identified papers that weren’t already included and found to be eligible, were included. Grey literature was also available for inclusion.

### Data extraction and analysis

For each article, the following data was extracted: author(s), year of publication, country of origin, study aims, study design, study population and key findings related to the research question. This was as per the JBI scoping review guidelines (Peters et al., 2020) and presented in tabular form (Table 1).

Included articles were independently assessed for methodical quality by reviewers (AF, CF). Quantitative studies were assessed using the National Heart, Lung and Blood Institute (NIH) quality assessment tool, (National Heart Lung and Blood Institute [NIH], Study Quality Assessment Tools, 2022) due to its ability to allow reviewers to focus on concepts regarding internal validity and its specificity for certain quantitative study designs. Qualitative studies were assessed using the JBI critical appraisal tool, (Briggs, 2022) due to its appropriateness for qualitative studies. Mixed methods studies were assessed using both tools. Articles were excluded based on article quality, as outlined in each tool.

The scoping review guidelines have evolved over the past two decades and Levac et al. (Levac et al., 2010) built on the framework outlined by Arksey and O’Malley (2005) to recommend the inclusion of a quality assessment in scoping reviews. The updated JBI guidelines exclude the use of a quality assessment. However, the authors considered that this assessment would assist in identifying strengths and weaknesses of included studies. Any differences in interpretation of the items were resolved by discussion with CG, allowing a final assessment of quality to be made.

## Results

### Search and inclusion results

**Fig. 1:**
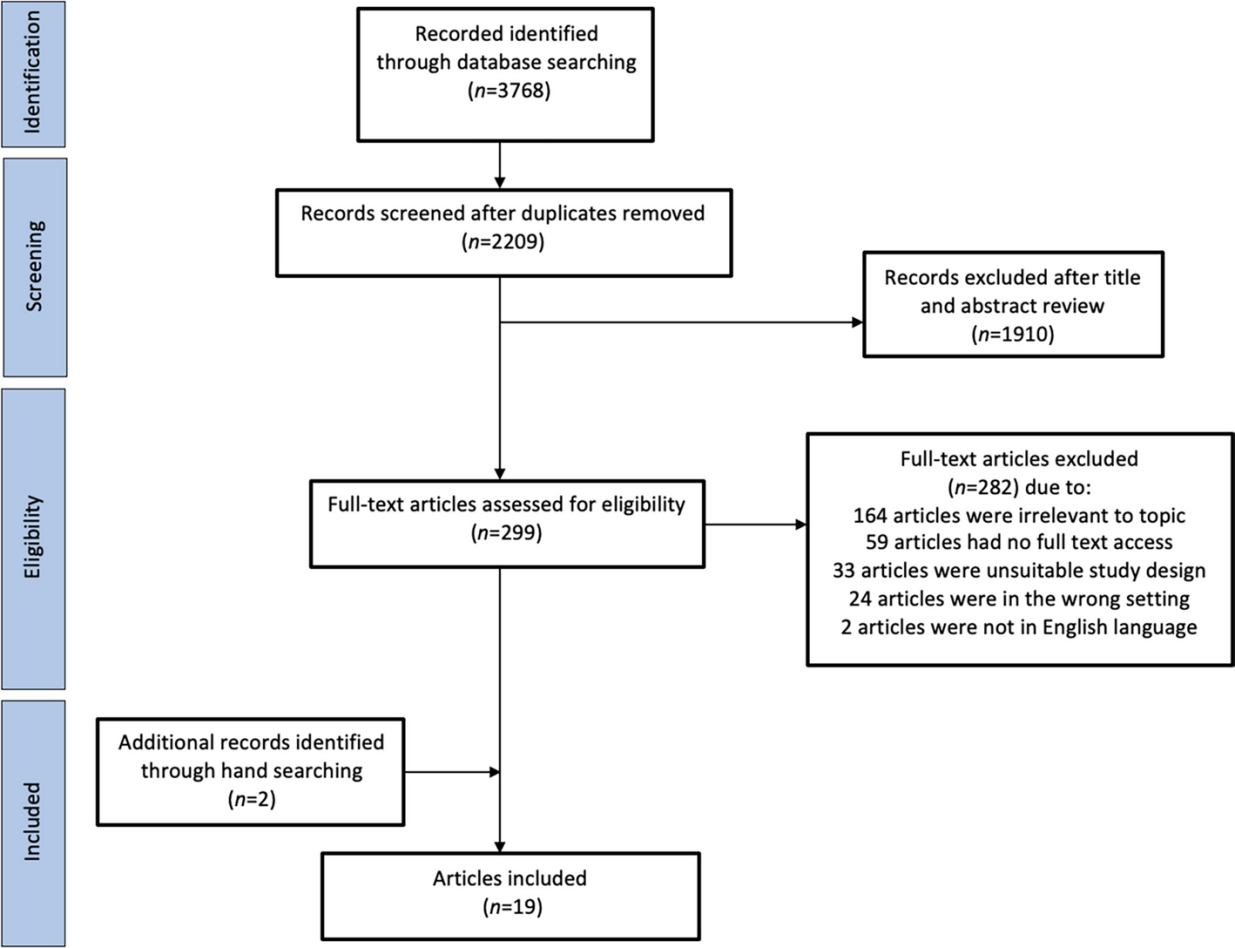
Flowchart of identification of eligible studies

A total of 2888 articles were identified in 2022. In 2023, a further 880 articles were identified. In total, 2209 articles were screened after the removal of duplicates (n=679). Figure 1 illustrates the search and study selection process. No new articles were identified for inclusion after full text screening, following re-running the search in 2023.

### Descriptive results

The 19 included articles covered SDL research conducted across 14 countries. The Netherlands (Berkhout et al., 2015, 2017; Frambach et al., 2012), Malaysia (Foong et al., 2021; Kek & Huijser, 2011; Ramamurthy et al., 2021) and the United States of America (Kastenmeier et al., 2018; Liu & Sullivan, 2021; Strowd et al., 2016) accounted for 3 studies each, and India (Bhandari et al., 2020; Devi et al., 2012), 2 studies. 16 studies sampled medical students (Berkhout et al., 2015, 2017; Bhandari et al., 2020; Devi et al., 2012; Foong et al., 2021; Frambach et al., 2012; Jouhari et al., 2015; Kastenmeier et al., 2018; Kek & Huijser, 2011; Kidane et al., 2020; Leatemia et al., 2016; Lee et al., 2010; Liu & Sullivan, 2021; Qaiser et al., 2020; Ramamurthy et al., 2021; Strowd et al., 2016), 2 utilised nursing students, (Cadorin et al., 2015; Pryce-Miller & Serrant, 2019) and 1 midwifery students. (Embo & Valcke, 2019) Studies were published in 15 different journals; “BMC Medical Education” was the most common with 3 articles (Foong et al., 2021; Kidane et al., 2020; Liu & Sullivan, 2021). The included research was relatively recent, with publications spanning a period from 2005 to 2022. Study designs were diverse, with qualitative studies most common (Berkhout et al., 2015, 2017; Bhandari et al., 2020; Embo & Valcke, 2019; Foong et al., 20121; Frambach et al., 2012; Jouhari et al., 2015; Kidane et al., 2020; Leatemia et al., 2016; Lee et al., 2010; Liu & Sullivan, 2021; Pryce-Miller & Serrant, 2019; Qaiser et al., 2020). A summary of results of the included studies is provided in Table 1.

**Table 1:** Data summary of included studies

Author/year	Country	Quality	Study aims	Study design	Study population	Key findings
Berkhout et al. (2015)	The Netherlands	JBI: Include	To identify factors that support or hamper medical students’ self-regulated learning (SRL) in a clinical context	Ground theory: semi-structured interviews	17 undergraduate medical students	SRL was influenced by goal setting, learning opportunities and facilities availability, expected outcomes, peers, learning atmosphere, engagement, patient interactions, autonomy and social relationships.
Berkhout et al. (2017)	The Netherlands	JBI: Include	To identify how medical students perceive the influence of other people in clinical settings on their SRL	Grounded theory: semi-structured interviews	14 undergraduate medical students	Residents were important in novice students’ SRL, through goal setting, learning experiences, encouraging self-reflection and feedback. Consultants and nurses were important for the learning environment and engagement. Experienced students were not as influenced by residents and these students needed greater autonomy and responsibility. Consultants had a more prominent role. Peers were important for students’ SRL.
Bhandari et al. (2020)	India	JBI: Include NIH: Fair	To assess the self-directed learning (SDL) abilities of first year Bachelor of Medicine, Bachelor of Surgery students.	Cross-sectional study: questionnaire	74 undergraduate medical students	Time management, evaluation of SDL, opportunities to participate and assistance with searching for resources would encourage SDL. Educators have multiple roles in SDL.
Cadorin et al. (2015)	Italy	NIH: Good	To evaluate the effect of different tutorial strategies offered to nursing students on their SDL abilities.	Cohort study: pre-post intervention	201 undergraduate nursing students.	High SDL scores were obtained by those learning in groups of peers composed of 4 students supervised by 1 clinical tutor.
Devi et al. (2012)	India	NIH: Fair	To compare the Self-Directed Learning Readiness (SDLR) among medical students who experienced the traditional curriculum with clinical exposure from the 2nd year of the course and among medical students who experienced a partially problem based curriculum	Cross-sectional study: questionnaire	240 undergraduate medical students	Students in the traditional curriculum had higher SDLR scores, than the hybrid curriculum. Early exposure clinical teaching, patient case presentations and clinician observation fostered SDL skills.
Embo and Valcke (2019)	Belgium	JBI: Include	To improve our understanding of regulating midwifery students’ learning by exploring factors that promote or inhibit the capacity to independently self-regulate learning during internships	Qualitative study: semi-structured focus groups	20 undergraduate midwifery students	Mentors on clinical placement were crucial for the development of their SRL skills. Students shared expectations and this was effective if based on the midwifery competency framework with corresponding learning goals, tasks and assessment criteria. Students required assistance in finding learning opportunities and in developing autonomy. Peer interactions enhanced their SRL.
Foong et al. (2021)	Malaysia	JBI: Include	To explore the characteristics of high performing medical students from the SRL perspective to gain a better understanding of the application of SRL for effective learning	Qualitative study: guided reflective journal and semi-structured interviews	21 undergraduate medical students	High performing students set high-achieving goals, could search for learning resources effectively, used mind maps regularly and had good time management.
Frambach et al. (2012)	Hong Kong, the Middle East and the Netherlands	JBI: Include	To investigate how students’ cultural backgrounds impact on SDL in problem-based learning (PBL) and how this impact affects students	Qualitative study: semi-structured, in-depth interviews	88 undergraduate medical students	Middle Eastern students were unable to find appropriate information and wanted more guidance. Hong Kong students attached greater value to tutorials that were facilitated by experts. Students valued PBL for assessment preparation.
Jouhari et al. (2015)	Iran	JBI: Include	To explore the views of medical students on the factors affecting SRL	Qualitative study: semi-structured, in-depth interview	19 undergraduate medical students	Students were motivated and learned from their peers. Instructors had positive impact on SRL if they were motivated, responsible, had good communication, engaged, had effective lesson-planning, teaching methods and provision of feedback. Students' SRL could be facilitated by having opportunities for learning, appealing environment, suitable equipment, support services and applicable curriculum. An inability to make daily schedules or define goals can inhibit SRL.
Kastenmeier et al. (2018)	United States of America	NIH: Good	To determine the impact of Individual Learning Plans (ILPs) on educational outcomes and acquisition of SDL skills	Cross sectional study: Questionnaire	394 undergraduate medical students	SDL exercises, such as the ILP, had a strong education value, lead to improved education outcomes and rated high by students. 'Professor Rounds' were a more effective method of developing SDL then ILP.
Kek and Huijser (2011)	Malaysia	NIH: Fair	To study the influence and impact of personal, family and learning and teaching environments on students’ approaches to learning and examine whether they adopt a deep or surface approach, and how that relates to the outcome measure of SDLR	Cross-sectional study: questionnaire.	392 undergraduate medical students and 32 teachers	Peer support within the university environment, engagement with peers within the classroom, deep learning approaches and educators who employed a student-focused teaching approach, improved SDL.
Kidane et al. (2020)	Ethiopia	JBI: Include NIH: Fair	To explore preclinical students following a hybrid curriculum in Ethiopia experiences to SDL and the support of several learning activities from the curriculum on their SDL	Mixed methods: cross-sectional questionnaire and focus group discussions	62 undergraduate medical students	PBL tutorial discussion and tutors had a high influence on students' SDL, whereas lectures and tests had a low influence. Support from educators, particularly during Year 1, was critical.
Leatemia et al. (2016)	Indonesia	JBI: Include NIH: Fair	To identify the student’s readiness to perform SDL and the underlying factors influencing it on the hybrid PBL curriculum	Mixed methods: cross-sectional questionnaire and focus group discussions	1178 undergraduate medical students	Students' SDL was influenced by tutors, PBL methods, peers, assessments, time management, relevant content to their future career and learning facilities and atmosphere. Increased numbers of students in a tutorial, increased student engagement. Tutors negatively impacted SDL if they interrupted students' discussion, limited discussion topics by providing teaching or if instructions were unclear.
Lee et al. (2010)	Canada	JBI: Include NIH: Fair	To investigate the influence of the case-oriented problem-stimulated curriculum on students' SDL	Mixed methods study: questionnaire and focus group interviews	119 undergraduate medical students	Tutorial discussion and unit/case objectives had a weak correlation with SDL ability. Lectures were not correlated. Expectations of peers and group dynamics influenced students' SDL. SDL improved through active learning, personal responsibility, critical analysis, increasing sociability and engagement of peers.
Liu and Sullivan (2021)	United States of America	JBI: Include	To determine whether and how students' descriptions of experiences and social contexts in clinical training could inform and extend current models of SDL	Interpretivist approach: semi-structured interviews	15 undergraduate medical students	Engagement in SDL was increased with patient care activities and greater autonomy. Finding learning resources, goal setting collaborative peer learning, feedback, support, cognitive strategies such as note-taking, use of question banks and self-quizzing were critical to be effective in SDL. Impression management, or how students would be perceived to look or perform, inhibited SDL.
Pryce-Miller and Serrant (2019)	United Kingdom	JBI: Include	To explore how pre-registration nursing students experience self-direction in learning and how they make meaning of their experience	Phenomenological approach: semi-structured interviews	6 undergraduate nursing students	Educators needed to be supportive, guiding and encouraging. They need to be creative and flexible in their strategies to address individual learning needs.
Qaiser et al. (2020)	Pakistan	JBI: Include	To explore supportive factors that regulate SRL of medical students	Qualitative study: semi-structured interviews	15 undergraduate medical students	Goal setting, time management, reflection and finding resources for learning are crucial for SRL.
Ramamurthy et al. (2021)	Malaysia	NIH: Fair	To measure the orientation of medical students toward lifelong learning and to determine the types of SDL activities that contribute toward lifelong skills	Cross-sectional study: questionnaire	329 undergraduate medical students.	PBL, flipped classroom and projects were more effective in promoting SDL by the pre-clinical students. Clinical posting, electives, guided reading and portfolios were more effective for the clinical students.
Strowd et al. (2016)	United States of America	NIH: Fair	To define medical student goals in the neurology clerkship and explore the association between goal setting and student performance, clerkship satisfaction, SDL and interest in neurology	Cross-sectional study: questionnaire	440 undergraduate medical students	Students who set goals whilst on clerkships observed tendency toward SDL.

### Quality assessment results

Quality assessment using the NIH quality assessment tool (NIH, 2022) revealed 3 studies to be of ‘good’ overall quality (Cadorin et al., 2015; Kastenmeier et al., 2018; Strowd et al., 2016) and 7 studies to be of ‘fair’ quality (Bhandari et al., 2020; Devi et al., 2012; Kek & Huijser, 2011; Kidane et al., 2020; Leatemia et al., 2016; Lee et al., 2010; Ramamurthy et al., 2021). Quality assessment utilising the JBI tool (Briggs, 2022) revealed all 13 studies eligible for inclusion. Refer to Tables 5 and 6.

### Factors associated with improving the student learning experience

Several themes were identified within the investigated studies. These were: curricular elements, such as specific learning activities and opportunities (n=16); environment, including atmosphere, access to resources, support, and class ratios (n=11); educator influence for engaging, motivating and facilitating students and SDL (n=8); impact of peers in engagement and support (n=7); and clinical placement experiences (n=4). These themes are outlined in Table 2.

**Table 2:** Identified themes of included studies

Author/year	Theme
Berkhout et al. (2015)	Curricular elements Environment Impact of peers Clinical placement experiences
Berkhout et al. (2017)	Clinical placement experiences
Bhandari et al. (2020)	Curricular elements Environment Educator influence
Cadorin et al. (2015)	Environment
Devi et al. (2012)	Curricular elements Clinical placement experiences
Embo and Valcke (2019)	Curricular elements Environment Impact of peers Clinical placement
Foong et al. (2021)	Curricular elements Environment
Frambach et al. (2012)	Curricular elements Environment Educator influence
Jouhari et al. (2015)	Curricular elements Environment Educator influence Impact of peers
Kastenmeier et al. (2018)	Curricular elements
Kek and Huijser (2011)	Curricular elements Educator influence Impact of peers
Kidane et al. (2020)	Curricular elements Educator influence
Leatemia et al. (2016)	Curricular elements Environment Educator influence Impact of peers
Lee et al. (2010)	Curricular elements Environment Impact of peers
Liu and Sullivan (2021)	Curricular elements Environment Educator influence Impact of peers
Pryce-Miller and Serrant (2019)	Educator influence
Qaiser et al. (2020)	Curricular elements Environment
Ramamurthy et al. (2021)	Curricular elements Clinical placement experiences
Strowd et al. (2016)	Curricular elements

## Discussion

Previous research has established that personal characteristics of students can impact on SDL (Guglielmino, 1977; Slater et al., 2017; Yuan et al., 2012). Authors have highlighted relationships between cognition, motivation and behaviour, and key nursing capabilities. These include the ability to engage in reflective practice, problem-solve, and develop clinical reasoning skills (Kuiper et al., 2010). The aim of this review was to explore the drivers that improve the student learning experience in undergraduate clinical science programs that utilise SDL.

This review has found multiple curricular, environmental, and external factors that can influence the student learning experience in SDL. A focus on stimulating positive influential factors, may allow academics in future curriculum development, to ensure that SDL is implemented effectively and appropriately. This ensures students are developing the necessary skills to be lifelong learners and are successful in their future health profession career.

### Curricular elements

Goal setting (Berkhout et al., 2015; Embo & Valcke, 2019; Foong et al., 2021; Jouhari et al., 2015; Liu & Sullivan, 2021; Qaiser et al., 2020; Strowd et al., 2016) and time management skills (Bhandari et al., 2020; Foong et al., 2021; Jouhari et al., 2015; Leatemia et al., 2016; Qaiser et al., 2020) were outlined as important drivers for improving the student learning experience of SDL. This is supported by research by Douglass & Morris (2014) whereby students outlined goal setting and time management skills as facilitators for SDL, and a lack of motivation as a barrier. ‘Motivation’ and ‘self-efficacy’ are key personality characteristics that lead to higher SDL scores (Berkhout et al., 2015, 2017; Guglielmino, 1977). Students need to be motivated to identify their learning needs and have the drive and belief in their process of developing strategies to address those gaps. Mastery of these essential study skills is key for students’ development of SDL and should be considered when developing curriculum, particularly in the early levels.

Students require adequate access to learning opportunities for SDL, (Berkhout et al., 2015; Bhandari et al., 2020) such as regular events where students can actively participate, including debates, group discussions, seminars, and quizzes (Bhandari et al., 2020). These opportunities were largely influenced by personal, contextual, and social factors, some of which could hamper students’ ability to perceive the learning activity as specific to SDL (Berkhout et al., 2015). It was also highlighted that students were more engaged in SDL if it was an assessed activity, they could anticipate the expected outcomes, (Berkhout et al., 2015; Embo & Valcke, 2019; Frambach et al., 2012; Leatemia et al., 2016) and it was relevant to their future profession (Bhandari et al., 2020; Jouhari et al., 2015; Leatemia et al., 2016). Previous research has indicated that classes with compulsory attendance requirements and clear assessment structures facilitated SDL (Douglass & Morris, 2014). Whilst there is a component of independence and autonomy in development of SDL, the role of educators and peers in the learning process is important.

Activities involving patient care, note-taking, question banks, self-quizzing, concept maps, reflection, flipped classroom, projects, clinical postings, electives, guided reading, individual learning plans, portfolios, and problem-based learning (PBL) were highlighted as promoting SDL (Foong et al., 2021; Kastenmeier et al., 2018; Kidane et al., 2020; Leatemia et al., 2016; Lee et al., 2010; Liu & Sullivan, 2021; Qaiser et al., 2020; Ramamurthy et al., 2021). Nursing students who engaged in a curriculum incorporating PBL were shown to have significant improvements in critical thinking skills, deduction, evaluation and metacognitive awareness. This was achieved through increasing students’ involvement in learning as PBL allows students to learn whilst engaging in meaningful and ‘real world’ problems (Choi et al., 2014; Gholami et al., 2016). However, whilst activities such as PBL have a high influence on independent learning, (Kidane et al., 2020) lectures and tests deter students’ SDL ability (Kidane et al., 2020; Lee et al., 2010). This was contrasted by research by Devi et al. (2012) who found that students who participated in a traditional, lecture-based curriculum scored higher on the SDL readiness scale, than those who experience a hybrid curriculum, incorporating PBL. Furthermore, educators who employed a student-focused approach and actively engaged students were most effective at improving students’ SDL (Kek & Huijser, 2011). This is largely an andragogical and constructivist approach to teaching (Biggs & Tang, 2011; Knowles, 1975). However, earlier research noted that a teacher-centric model should be utilised in the foundational stages of their education, in order for students to develop an understanding of the principles of SDL (Iwasiw, 1987; Levett-Jones, 2005). Knowles et al. (1998) states that both educators and students must be familiar with the concept of SDL and have the tools required to employ self-directed learning strategies. This suggests that didactic instruction must occur initially for students to successfully engage (Levett-Jones, 2005). Inconsistency throughout the evidence, potentially illustrates that the implementation of SDL within curriculum has its challenges and there is a need for a more standardised approach.

Whilst inconsistencies may exist amongst the literature in regard to execution within curriculum, what is clear are the curricular elements which the influence student learning and engagement. Students need to be skilled in time management and goal setting and have regular access to learning opportunities that facilitate SDL. This includes PBL and patient care activities. Students also hold greater purpose in the learning activity if it’s linked to assessment. Such activities often promote active engagement and involvement, allowing students to be motivated in their learning and further develop SDL skills.

### Environment

The learning environment needs to have an appealing atmosphere and students need to have the access to appropriate equipment (Berkhout et al., 2015; Jouhari et al., 2015; Leatemia et al., 2016; Lee et al., 2010) and learning resources (Berkhout et al., 2015; Bhandari et al., 2020; Embo & Valcke, 2019; Foong et al., 2021; Frambach et al., 2012; Leatemia et al., 2016; Liu & Sullivan, 2021; Qaiser et al., 2020). A lack of access to technology has previously been found to impede the SDL process (Douglass & Morris, 2014). A fundamental component of SDL is the ability to identify resources for learning, (Knowles, 1975) however a lack of availability of resources could hamper this stage in the development process.

Higher SDL ability was reported when students worked in small groups with their peers and an educator, rather than one-on-one with an educator (Berkhout et al., 2015; Cadorin et al., 2015; Leatemia et al., 2016). This may be due to the degree of autonomy and adaptability students can develop in a more independent setting (Berkhout et al., 2015; Embo & Valcke, 2019; Liu & Sullivan, 2021). Small class sizes are supported within literature (Douglass & Morris, 2014) and can also promote students’ critical thinking, shared experiential learning, and exploration (Yuan et al., 2012). Madhavanprabhakaran et al. (2013) outlined nursing students preferred a 4:1 student to educator ratio in clinical learning. This suggests that an educator’s involvement may hamper students’ development of SDL skills by not creating space for students to take initiative, recognise their learning needs, and research resources for learning themselves, (Knowles, 1975) or with the assistance of peers.

Student support services also enhanced engagement in SDL (Jouhari et al., 2015). Research conducted by Liu and Sullivan (2021) outlined students were often inhibited by a fear of how they would appear to educators or peers, creating pressure and anxiety amongst students. Availability and access to appropriate support services, including university administration, can better support students through their learning process and within the learning environment.

There are various elements which impact on student learning and engagement in regard to SDL. To facilitate optimal environments which encourage the development of SDL in students, a wide range of factors must be considered. It is crucial to establish learning environments that are appealing and supportive. Students require adequate access to learning resources and equipment to ensure there is opportunity to develop SDL. Consideration should be made for the student and educator ratios within learning environments to ensure students are given appropriate opportunity for autonomous learning.

### Educator influence

Educators were found to be key in students’ development of SDL. Whilst fundamental to the role was the facilitation of curricular activities, the most significant impact of the educator was through motivating students, mentorship, evaluation, and provision of feedback (Bhandari et al., 2020; Frambach et al., 2012; Jouhari et al., 2015; Kek & Huijser, 2011; Kidane et al., 2020; Leatemia et al., 2016; Liu & Sullivan, 2021; Pryce-Miller & Serrant, 2019). Motivating students to be proactive in and outside of class has been found to be of particular importance, such as through encouraging class attendance, participation, and networking (Douglass & Morris, 2014). Walker et al. (2014) outlined that good role modelling facilitates learning. Conversely, educators who were poor role models hampered the learning experience of students and negatively affected morale and perception of the nursing discipline. In order for students to effectively develop SDL skills, educators must role model good behaviours to motivate students to engage in activities which promote SDL. Consequently, students may have greater well-being and encouragement to enter the profession.

Educators need to provide clear instructions regarding the learning activity for the promotion of SDL (Jouhari et al., 2015; Leatemia et al., 2016). Leatemia et al. (2016) noted that educators needed to not limit students’ discussion by interrupting or providing teaching. Educators should encourage independence in learning and adopt teaching approaches that promote active learning (du Toit-Brits, 2019). Students should be empowered to be confident to solve problems, take initiate to self-identify their learning needs and formulate learning goals and strategies.

Cultural factors can also impact on a students’ engagement with educators, readiness to engage in SDL and their SDL abilities. Frambach et al. (2012) outlined students from Hong Kong attached higher value to SDL tutorials if they were led by expert clinicians. This was attributed to a teacher-centred secondary education and society’s respect for hierarchy. Furthermore, Frambach et al. (2012) identified that Middle Eastern students outlined a culturally determined focus on tradition and society-driven respect for the ‘old ways’. This made students more inclined to be resistive to SDL, in preference for traditional curriculum. Acknowledgement of these impacts when designing and implementing learning activities is essential to ensure student success in SDL. This is supported by Pryce-Miller and Serrant (2019) who found that educators need to be creative and flexible in addressing individual learning needs. Educators are a fundamental influencing aspect on the implementation of SDL and need to have awareness of the roles they adopt in fostering students’ SDL development.

### Impact of peers

Peers have an essential role in promoting SDL. Students were able to collaborate and engage with their peers, were motivated and supported by their peers, and learned from their experiences (Berkhout et al., 2015; Embo & Valcke, 2019; Jouhari et al., 2015; Kek & Huijser, 2011; Leatemia et al., 2016; Lee et al., 2010; Liu & Sullivan, 2021). Williams et al. (2015) highlighted ‘near peer teaching’ programmes assisted paramedicine students’ development of skills, knowledge and confidence. Peer-assisted learning programmes have also been found to improve students’ ability to provide feedback, skill performance in examinations, and sense of competency through self-assessment against their peers (Silbert & Lake, 2012; Walker et al., 2014). Working collaboratively with peers enabled nursing students to appreciate the importance of effective teamwork (Walker et al., 2014). This contrasts with research by Berkhout et al. (2015) who highlighted that students’ SDL was at times hampered by the presence of peers due to reducing learning opportunities. However, this wasn’t a consistent finding, with some students outlining working with peers provided opportunities to discuss doubts and ask questions that they didn’t feel comfortable raising with the educator. Research by O’Mara et al. (2014) outlined that nursing students utilised their peers for support in challenging clinical environments. Social cognitive theory informs that learning is significantly affected by interactions between students, and students and educators. Learning in a social context can also improve motivation for learning outcomes (Uijl et al., 2017). Peer interaction can also be through proactive involvement in student organisations, networking or study groups (Douglass & Morris, 2014). Social interaction is key in educational success. Most students need to feel part of a group to desire to learn.

The impact of peers on the student learning experience is multi-faceted. Overwhelming, students encouraged the development of SDL through collaboration, motivation, experiential learning, and active engagement. Students also utilised each other for support through their learning. Modern curricular approaches should reflect the positive influential involvement of peers in the learning process, the role of peers in the well-being of others, and the development of SDL skills. This may include opportunities for collaborative learning and peer assessment.

### Clinical placement experiences:

Students on clinical placement are ideally positioned to utilise SDL principles. Berkhout et al. (2015) reported that student interactions with patients promoted SDL. Furthermore, SDL was enhanced when students participated in clinical placement early in their education (Devi et al., 2012; Ramamurthy et al., 2021). This is supported by research which highlighted students found placement valuable in gaining practice and experience, and promoted learning and preparedness for professional practice (Douglass & Morris, 2014; Flott & Lindon, 2016). The case-based learning also allowed for deeper learning and therefore critical thinking, (McLean, 2016) a key result of competency in SDL.

The role of clinical placement supervisors, such as hospital medical residents, was outlined as crucial for guiding students through goal setting, self-reflection, support, and content knowledge (Berkhout et al., 2017; Embo & Valcke, 2019). This is most effective when these elements aligned with relevant assessment and competency frameworks (Embo & Valcke, 2019). Medical residents did not have as significant an impact regarding these components when students were ‘experienced’ in the clinical setting (Berkhout et al., 2017). It could be considered that these students have had greater opportunity to develop SDL skills due to previous exposures. Furthermore, an increase in age of students is associated with higher SDL scores (Cadorin et al., 2015; Meng et al., 2019; Slater et al., 2017).

The role of peers was noted to be invaluable whilst students were on clinical placement (Berkhout et al., 2017). Peers provide a sense of safety when entering the clinical environment and introduced to new staff or presented with clinical challenges. Peers also influence the degree of learning students gained from clinical placement, as students can assess each other, assuring they had no learning needs. However, peers have been noted to be obstacle for learning when on clinical placement, particularly when students are placed together with a supervisor. A sense of competition can exist for the supervisor’s time and attention, in relation to patient care situations, and when practicing technical skills (Stenberg & Carlson, 2015).

Clinical placement is a highly beneficial learning activity to develop and enhance SDL abilities in students. Clinical supervisors need to be adequately prepared to facilitate students and have the resources to support students in their learning and development, particularly with more junior students.

### Limitations

Whilst attempting to include an extensive range of clinical science programs, some disciplines may have been unintentionally excluded. The majority of included literature was found to sample undergraduate medical students. Whilst the principle of SDL is relevant amongst all clinical science programs, this sample population may affect the generalisability of the results. Articles were excluded in languages other than English, to avoid the need for transcribing, which meant that some potentially relevant articles may not have been included. Furthermore, it was noted that there were various interpretations and definitions of SDL. Whilst, the search strategy attempted to include all of these, it may not be exhaustive. It was also noted that there are various SDL activities, such as PBL, that may not have fit the outlined inclusion criteria but may have been relevant to study.

## Conclusion

This review explored what is known about the drivers that improve the student learning experience in clinical science programs that utilise SDL. The recency of articles suggests an evolving interest in SDL within health professional education. This study revealed there are multiple factors which can impact on the students’ ability to effectively engage and be successful in SDL, including curriculum, impact of educators and peers, environment, and clinical placement. The utilisation of SDL as a learning technique requires adequate preparedness of educators, for students to successfully engage and develop SDL skills. These findings can inform curriculum development and implementation in clinical science programs, to better prepare future health professionals to be lifelong learners.

## Appendix

See in Tables 3, 4, 5 and 6.

**Table 3:** Search strategy as per MEDLINE (via OVID). Search was limited to human studies and full text

	Search Concept	keywords
#1	Undergraduate clinical science students	(student* OR degree OR bachelor OR pre-med* OR undergrad*) AND (clinical science OR health OR nurs* OR paramedic* OR allied health OR medic* OR dentist* OR osteopath* OR speech path* OR physio* OR physical therapy OR occupational therapy OR midwifery OR radiography OR medical imag* OR OT OR PT)
#2	Self-directed learning	((self-direct* OR independent OR student-direct* OR self-guid* OR self-regulat*) N2 (learn* OR stud*)) OR (self-learn* OR sdl OR self-stud*)
#3	Drivers in learning experience	(factor* OR element* OR component* OR motivat* OR driver*) AND (learn* experience* OR outcome* OR experience*)

**Table 4:** Search strategy as per Medline (via OVID) including text word searching, truncation and field-specific searches

1	(Clinical science or health or nurs* or paramedic* or allied health or medic* or dentist* or osteopath* or speech path* or physio* or physical therapy or occupational therapy or midwifery or radiography or medical imag* or OT or PT).mp. [mp=title, book title, abstract, original title, name of substance word, subject heading word, floating sub-heading word, keyword heading word, organism supplementary concept word, protocol supplementary concept word, rare disease supplementary concept word, unique identifier, synonyms, population supplementary concept word, anatomy supplementary concept word]
2	(Student* or degree or bachelor or pre-med* or undergrad*).mp. [mp=title, book title, abstract, original title, name of substance word, subject heading word, floating sub-heading word, keyword heading word, organism supplementary concept word, protocol supplementary concept word, rare disease supplementary concept word, unique identifier, synonyms, population supplementary concept word, anatomy supplementary concept word]
3	1 and 2
4	((self-direct* or independent or student-direct* or self-guid* or self-regulat*) adj2 (learn* or stud*)).mp. [mp=title, book title, abstract, original title, name of substance word, subject heading word, floating sub-heading word, keyword heading word, organism supplementary concept word, protocol supplementary concept word, rare disease supplementary concept word, unique identifier, synonyms, population supplementary concept word, anatomy supplementary concept word]
5	(Self-learn* or SDL or self-stud*).mp. [mp=title, book title, abstract, original title, name of substance word, subject heading word, floating sub-heading word, keyword heading word, organism supplementary concept word, protocol supplementary concept word, rare disease supplementary concept word, unique identifier, synonyms, population supplementary concept word, anatomy supplementary concept word]
6	4 or 5
7	(Factor* or element* or component* or motivat* or driver*).mp. [mp=title, book title, abstract, original title, name of substance word, subject heading word, floating sub-heading word, keyword heading word, organism supplementary concept word, protocol supplementary concept word, rare disease supplementary concept word, unique identifier, synonyms, population supplementary concept word, anatomy supplementary concept word]
8	(Learn* experience* or outcome* or experience*).mp. [mp=title, book title, abstract, original title, name of substance word, subject heading word, floating sub-heading word, keyword heading word, organism supplementary concept word, protocol supplementary concept word, rare disease supplementary concept word, unique identifier, synonyms, population supplementary concept word, anatomy supplementary concept word]
9	7 and 8
10	3 and 6 and 9

**Table 5:** National Heart, Lung and Blood Institute (NIH) quality assessment tool results

	Bhandari et al. (2020)	Cadorin et al. (2015)	Devi et al. (2012)	Kastenmeier et al. (2018)	Kek and Huijser (2011)	Kidane et al. (2020)	Leatemia et al. (2016)	Lee et al. (2010)	Ramamurthy et al. (2021)	Strowd et al. (2016)
1. Research question	No	Yes	Yes	No	Yes	Yes	Yes	Yes	Yes	Yes
2. Study population	Yes	Yes	Yes	Yes	Yes	Yes	Yes	Yes	Yes	Yes
3. Participation rate	Yes	Yes	Yes	Yes	Other	Yes	Other	Yes	Yes	Yes
4. Recruitment & eligibility	Yes	Yes	Yes	Yes	Yes	Yes	Yes	Yes	Yes	Yes
5. Sample size	No	No	No	No	No	No	No	Yes	Yes	No
6. Exposure(s) of interest	No	Yes	Yes	Yes	No	No	No	No	No	Yes
7. Timeframe	No	No	No	Yes	No	No	No	No	No	Yes
8. Levels of exposure	Yes	Yes	Yes	Yes	Yes	Yes	Yes	Yes	Yes	Yes
9. Exposure measures criteria	Yes	Yes	Yes	Yes	Yes	Yes	Yes	Yes	Yes	Yes
10. Repeated exposure assessment	No	Yes	No	Yes	No	No	No	No	No	No
11. Outcome measures	Yes	Yes	Yes	Yes	Yes	Yes	Yes	Yes	Yes	Yes
12. Blinding	Other	Yes	Other	Other	No	No	No	Other	No	No
13. Follow-up rate	Yes	Yes	Yes	Yes	Other	Yes	Other	Yes	Other	Yes
14. Statistical analyses	No	Yes	No	No	Other	No	No	No	No	Yes
15. Quality Rating (good, fair or poor)	Fair	Good	Fair	Good	Fair	Fair	Fair	Fair	Fair	Good

**Table 6:** Joanna Briggs Institute (JBI) critical appraisal tool results

	Berkhout et al. (2015)	Berkhout et al. (2017)	Bhandari et al. (2020)	Embo and Valcke (2019)	Foong et al. (2021)	Frambach et al. (2012)	Jouhari et al. (2015)	Kidane et al. (2020)	Leatemia et al. (2016)	Lee et al. (2010)	Liu and Sullivan (2021)	Pryce-Miller and Serrant (2019)	Qaiser et al. (2020)
1. Congruity between philosophical perspective and research methodology?	Yes	Yes	Unclear	Unclear	Unclear	Yes	Unclear	Unclear	Unclear	Unclear	Yes	Yes	Unclear
2. Congruity between methodology and research question?	Yes	Yes	Yes	Yes	Yes	Yes	Yes	Yes	Yes	Yes	Yes	Yes	Yes
3. Congruity between the methodology and the methods of data collection?	Yes	Yes	Yes	Yes	Yes	Yes	Yes	Yes	Yes	Yes	Yes	Yes	Yes
4. Congruity between methodology and data representation and analysis?	Yes	Yes	Yes	Yes	Yes	Yes	Yes	Yes	Yes	Yes	Yes	Yes	Yes
5. Congruity between methodology and interpretation?	Yes	Yes	Yes	Yes	Yes	Yes	Yes	Yes	Yes	Yes	Yes	Yes	Yes
6. Statement locating the researcher culturally/theoretically?	Yes	Yes	Yes	Yes	Yes	Yes	Yes	No	No	No	Yes	No	Yes
7. Influence of the researcher on the research, and vice- versa, addressed?	No	No	No	No	Yes	Yes	No	No	No	No	No	No	No
8. Participant adequately represented?	Yes	Yes	Yes	Yes	Yes	Yes	Yes	Yes	Yes	Yes	No	Yes	Unclear
9. Ethical according to current criteria or, evidence of ethical approval by an appropriate body?	Yes	Yes	Yes	Yes	Yes	Yes	Yes	Yes	Yes	Yes	Yes	Yes	Unclear
10. Conclusions drawn flow from the analysis, or interpretation, of the data?	Yes	Yes	Yes	Yes	Yes	Yes	Yes	Yes	Yes	Yes	Yes	Yes	Yes
11. Overall appraisal	Include	Include	Include	Include	Include	Include	Include	Include	Include	Include	Include	Include	Include
