# Correction: Self-directed learning and the student learning experience in undergraduate clinical science programs: a scoping review

**DOI:** 10.1007/s10459-024-10389-1

**Published:** 2024-11-25

**Authors:** Ashleigh Finn, Caitlin Fitzgibbon, Natalie Fonda, Cameron M. Gosling

**Affiliations:** 1 Department of Paramedicine, Monash University, Level 2, Building H, Peninsula Campus, McMahons Road, Frankston, VIC 3199 Australia; 2 School of Nursing, Midwifery & Paramedicine, Australian Catholic University, 115 Victoria Parade, Fitzroy, VIC 3065 Australia

**Correction to: Advances in Health Sciences Education**

10.1007/s10459-024-10383-7

In this article the affiliation details for Caitlin Fitzgibbon were incorrectly given as ‘Department of Paramedicine, Monash University, Level 2, Building H, Peninsula Campus, McMahons Road, Frankston VIC 3199, Australia’ but should have been ‘School of Nursing, Midwifery & Paramedicine, Australian Catholic University, 115 Victoria Parade, Fitzroy VIC 3065, Australia’.

The original article has been corrected.

